# Genetic variation in the functional *ENG* allele inherited from the non-affected parent associates with presence of pulmonary arteriovenous malformation in hereditary hemorrhagic telangiectasia 1 (HHT1) and may influence expression of *PTPN14*

**DOI:** 10.3389/fgene.2015.00067

**Published:** 2015-03-12

**Authors:** Tom G. W. Letteboer, Michael Benzinou, Christopher B. Merrick, David A. Quigley, Kechen Zhao, Il-Jin Kim, Minh D. To, David M. Jablons, Johannes K. P. van Amstel, Cornelius J. J. Westermann, Sophie Giraud, Sophie Dupuis-Girod, Gaetan Lesca, Jonathan H. Berg, Allan Balmain, Rosemary J. Akhurst

**Affiliations:** ^1^Helen Diller Family Comprehensive Cancer Center, University of California, San Francisco San Francisco, CA, USA; ^2^Department of Medical Genetics, University Medical Centre Utrecht Utrecht, Netherlands; ^3^Department of Clinical Genetics, University of Dundee Dundee, UK; ^4^Department of Surgery, University of California, San Francisco San Francisco, CA, USA; ^5^Department of Pulmonology, St. Antonius Hospital Nieuwegein, Netherlands; ^6^Department of Medical Genetics, Lyon University Hospital Lyon, France; ^7^Department of Biochemistry and Biophysics, University of California, San Francisco San Francisco, CA, USA; ^8^Institute of Human Genetics, University of California, San Francisco San Francisco, CA, USA; ^9^Department of Anatomy, University of California, San Francisco San Francisco, CA, USA

**Keywords:** endoglin, ACVRL1, PTPN14, EMILIN2, lung, arteriovenous malformations

## Abstract

HHT shows clinical variability within and between families. Organ site and prevalence of arteriovenous malformations (AVMs) depend on the HHT causative gene and on environmental and genetic modifiers. We tested whether variation in the functional *ENG* allele, inherited from the unaffected parent, alters risk for pulmonary AVM in HHT1 mutation carriers who are *ENG* haploinsufficient. Genetic association was found between *rs10987746* of the wild type *ENG* allele and presence of pulmonary AVM [relative risk = 1.3 (1.0018–1.7424)]. The *rs10987746*-C at-risk allele associated with lower expression of *ENG* RNA in a panel of human lymphoblastoid cell lines (*P* = 0.004). Moreover, in angiogenically active human lung adenocarcinoma tissue, but not in uninvolved quiescent lung, *rs10987746*-C was correlated with expression of *PTPN14* (*P* = 0.004), another modifier of HHT. Quantitative TAQMAN expression analysis in a panel of normal lung tissues from 69 genetically heterogeneous inter-specific backcross mice, demonstrated strong correlation between expression levels of *Eng, Acvrl1*, and *Ptpn14* (*r*2 = 0.75–0.9, *P* < 1 × 10^−12^), further suggesting a direct or indirect interaction between these three genes in lung *in vivo*. Our data indicate that genetic variation within the single functional *ENG* gene influences quantitative and/or qualitative differences in *ENG* expression that contribute to risk of pulmonary AVM in HHT1, and provide correlative support for *PTPN14* involvement in endoglin/ALK1 lung biology *in vivo*. *PTPN14* has been shown to be a negative regulator of Yap/Taz signaling, which is implicated in mechanotransduction, providing a possible molecular link between endoglin/ALK1 signaling and mechanical stress. *EMILIN2*, which showed suggestive genetic association with pulmonary AVM, is also reported to interact with Taz in angiogenesis. Elucidation of the molecular mechanisms regulating these interactions in endothelial cells may ultimately provide more rational choices for HHT therapy.

## Introduction

Hereditary Hemorrhagic Telangiectasia (HHT) is an autosomal dominant disorder resulting from loss of function mutations predominantly in *ENG* (HHT type 1) or *ACVRL1 aka ALK1* (HHT type 2) (Shovlin, 2010; Faughnan et al., 2011). These genes encode cell surface receptors which are components of the TGF-β/BMP signal transduction pathways active predominantly in endothelial cells. Endoglin (*ENG*) is a trans-membrane glycoprotein similar to the TGF-β type III receptor and ALK1 (*ACVRL1*) is a transmembrane serine threonine kinase TGF-β/BMP type I receptor (Lebrin et al., 2004; Lopez-Novoa and Bernabeu, 2010; Pardali et al., 2010; Cunha and Pietras, 2011). Affected HHT patients typically present during adolescence with muco-cutaneous telangiectases and frequent severe episodes of epistaxis (Shovlin, 2010; Faughnan et al., 2011). Telangiectases result from abnormal vascular remodeling, leading to capillary breakdown and consequent shunting of arterioles directly into venules with no intervening capillary bed (Guttmacher et al., 1995). Blood vessel walls of lesions are dilated and weak, leading to frequent hemorrhage. Epistaxis and bleeding from gastrointestinal tract telangiectases can result in severe anemia and the need for frequent blood transfusions. HHT is highly penetrant, but with age dependent expression of different clinical features (Shovlin, 2010; Faughnan et al., 2011).

In addition to telangiectases, approximately half of HHT patients develop more serious complications, namely visceral AVMs that can occur anywhere but are mostly found in lung, brain, and liver. AVMs also involve shunting of blood directly from artery to vein. Large AVMs lead to local hypoxia and are prone to hemorrhage. Brain AVMs are often asymptomatic, but can cause seizures or hemorrhage. Pulmonary AVMs are at high risk of thromboembolism, resulting in thrombotic stroke or cerebral abscess. One of the features of HHT is a large range in clinical severity and prevalence of AVMs (Letteboer et al., 2006; Lesca et al., 2007; Sabba et al., 2007). Some phenotypic variability can be accounted for by genetic heterogeneity, for example lung AVMs are more common in HHT1, whereas hepatic AVMs are more prevalent in HHT2 (Letteboer et al., 2006; Lesca et al., 2007; Sabba et al., 2007). Nevertheless, the large intra-familial range in clinical severity suggests that local environmental stresses, such as wounding and/or inflammation (van Laake et al., 2006; Park et al., 2009; Garrido-Martin et al., 2014) and/or germline genetic modifiers (Benzinou et al., 2012; Kawasaki et al., 2014), may contribute to precipitation of telangiectases or AVMs. Localized somatic mutation (Akers et al., 2009; McDonald et al., 2014) might also contribute to the risk of clinical progression, although this has not been definitively tested. Identification of disease-modifying germline genetic variants may provide a deeper molecular understanding of the process of AVM formation that might be harnessed for therapeutic design purposes.

In order to uncover possible molecular mechanisms that contribute to pulmonary AVMs, we previously undertook a screen for genetic loci that associate with pulmonary AVM prevalence within the HHT population (Benzinou et al., 2012). As TGF-β1 is known to bind both endoglin and ALK1 and attenuate or activate signaling downstream of ALK1, we hypothesized that genetic modifiers of TGF-β1 (*Tgfbm's*) may influence the vascular outcome of HHT. We therefore screened genes within human loci syntenic to mouse *Tgfbm2* and *Tgfbm3*, both suppressors of a lethal prenatal vascular dysplasia observed in some *Tgfb1-/-* mice (Benzinou et al., 2012; Kawasaki et al., 2014). We also screened for genetic association within 72 additional human genes, genome-wide, that encode components of the TGF-β or BMP signaling pathways, or that had been implicated in regulating or being regulated by TGF-β or BMP (Benzinou et al., 2012). While we found only one gene, *PTPN14*, encoding protein tyrosine phosphatase, non-receptor type 14 (PTPN14), that fit our stringent genetic criteria for being a modifier of pulmonary AVM incidence in HHT (Benzinou et al., 2012), the current study focuses on weaker but still instructive genetic associations. We also provide further correlative evidence that *PTPN14* interacts with *ENG* in the lung *in vivo*.

## Methods

### HHT human subjects

Study protocols were approved by local ethics committees at all institutions, and informed consent was obtained from each subject before participation in the studies. Initially, 649 Dutch Caucasians and 72 Dutch Antillean Blacks were genotyped. These were selected from a panel consisting of probands and family members from a total of 137 different families screened for HHT. All manifestations of HHT were recorded for both probands and family members. The clinical diagnosis of HHT was established according to the Curaçao criteria (Faughnan et al., 2011), which are clinical features of epistaxis, multiple telangiectases, visceral AVMs and a first-degree relative with HHT. A diagnosis of HHT is considered definitive if three criteria are present, possible if two criteria are present and unlikely if fewer than two criteria are present. Mutation carrier status was confirmed by molecular analysis. Most probands and family members were screened for visceral manifestations at St Antonius Hospital, which specializes in the diagnosis and treatment of HHT. Screening for the presence of pulmonary AVMs was performed routinely by chest radiography and by measuring partial oxygen tension in arterial blood and, if abnormal, followed by the 100% oxygen right-to-left shunt test. Based on this analysis, patients with suspected pulmonary AVMs were offered conventional angiography, digital subtraction angiography of the pulmonary arteries or computed tomography (CT) of the chest. In some patients, pulmonary AVMs were diagnosed using high-resolution CT and/or contrast trans-thoracic echocardiography.

HHT clinical diagnosis in the French cohort was also established according to the Curaçao criteria. Mutation carrier status was confirmed by molecular diagnosis (heteroduplex analysis or denaturing high performance liquid chromatography and sequencing). The 222 French patients included in the present study all carried a mutation in either *ENG* (*n* = 76) or *ACVRL1* (*n* = 146), and 43% of them had pulmonary AVMs (74% for HHT1 patients and 27% for HHT2 patients). Familial structures included 111 singletons, 40 duos, 2 trios, 5 quartets, and 1 family with 5 individuals. The diagnosis of pulmonary involvement was made either in patients presenting symptoms (for example, dyspnoea and cyanosis) or complications (mainly brain abscess), or in asymptomatic HHT patients who underwent screening using contrast trans-thoracic echocardiography, chest radiograph and/or oxygen shunt test as described. Screening for pulmonary AVMs was also recommended to asymptomatic patients and accepted by a majority of them (Lesca et al., 2007). The “no-pulmonary AVM” cohort should thus be considered as either negative for pulmonary AVMs or having only small clinically-insignificant pulmonary AVMs at the time of assessment.

### Lymphoblastoid cell lines

Affymetrix gene expression data for 61 human lymphoblastoid cell lines derived from blood samples from Utah residents of Northern and Western European Ancestry from the CEPH collection (CEU) (Cheung et al., 2005), were downloaded from Gene Expression Omnibus (GEO), and matching genotype data for *rs10987746* was downloaded from the International HapMap Project website. Expression levels of *ENG* and *PTPN14* were compared by *rs10987746* genotype. Since the *rs10987746-C/C* genotype was unusually underrepresented in this panel (*n* = 7), and showed no statistically significant difference in expression from the *rs10987746*-*T/C* genotype, these two genotypes were pooled and their combined expression levels compared to that of the *rs10987746*-*T/T* genotype. *P-values* (*ANOVA*) were calculated using Tukey's HSD in R Development Core Team (2011).

### Human lung cancer subjects

All samples were obtained from patients with informed consent under the IRB approval (ID: 10-03352) granted by the Committee on Human Research at the University of California, San Francisco (UCSF). Paired RNA and DNA samples from human primary lung adenocarcinomas and uninvolved “normal” lung tissue were obtained from patients undergoing tumor debulking surgery at UCSF. Each tumor sample consisted of at least 50% tumor cells.

### Microarray expression analysis of paired normal and adenocarcinoma lung tissue from lung cancer patients

RNA was extracted with Trizol followed by DNAse I treatment and purification (RNeasy RNA purification kit, Qiagen). The purified RNA was quantified with Nanodrop and QC checked by Agilent Bioanalyzer. Gene expression was measured with the Illumina Human Whole Genome 6 2.0 array (Illumina Inc., San Diego, CA, USA). Sample preparations, hybridization and scanning were performed according to manufacturer's instructions. Briefly, 200 ng of total RNA was prepared to make cRNA by using Illumina TotalPrep RNA Amplification Kit (Ambion). First-strand complementary DNA was generated with T7 oligo (dT) primer and ArrayScript and then second cDNA was also made with DNA polymerase. Biotin-NTP with T7 enzyme mixes was used to make biotinylated cRNAs. The labeled cRNAs were purified, quantified and checked again using the Bioanalyzer. The labeled cRNA target was used for hybridization and scanning according to Illumina Human Whole Genome 6 2.0 array protocol. Probes with present/absent call ≥0.05 assessed by Illumina Beadstudio software, were marked absent. Raw microarray data were quantile normalized and log2-transformed. Statistical analysis was performed with R version 2.13 (R Development Core Team, 2011).

### Human SNP selection

Gene-centric tag-SNPs were chosen on the basis of being non-synonymous coding polymorphisms, potential splice site variants or inferred to disrupt important gene regulatory sites (that is, microRNAs and transcription factor binding sites, and so on) using the UCSC genome server. We further looked for SNPs located in highly conserved regions in the 3′ UTR. This first set of SNPs was complemented by tag-SNPs using a tag approach with the HapMap phase II database (http://www.hapmap.org) by “force including” the first set of variants using the Haploview Tagger and by searching for additional tag-SNPs. Selected SNPs were estimated to give ~85% coverage of any chosen gene.

### Human genotyping and quality control

We previously screened genetic variants for association with the presence of pulmonary AVM in HHT patients from 137 Dutch HHT families. The first screen included 649 Dutch individuals of northern European descent and 72 Afro–Caribbean individuals from the Dutch Antilles. In addition to screening SNPs within *TGFBM2* (Benzinou et al., 2012) and *TGFBM3* (Kawasaki et al., 2014), we screened tag-SNPs (*n* = 443) that covered 72 “candidate genes” selected on the basis of their involvement in TGF-β or BMP signaling and/or their responses to TGF-β. We used a modification of the transmission disequilibrium test (TDT), namely Gamete Competition (GC) (Lange et al., 2001, 2005) to screen for genetic association with the presence *vs*. absence of pulmonary AVM in HHT mutation carriers. GC was selected over TDT because of the large, multigenerational structure of families within this cohort. In addition to the previously published genetic associations (Benzinou et al., 2012; Kawasaki et al., 2014) 17 of the 443 SNPs tagging candidate genes showed nominal evidence of over-transmission to pulmonary AVM+ in Dutch HHT patients (*P* < 0.05, GC test), and were genotyped in an additional 108 northern European Dutch individuals (Extension study). Genotyping of the first Dutch cohort was performed using 750-ng labeled genomic DNA hybridized to a custom Illumina chip. Genotyping for the Dutch extension and French replication studies was performed using Sequenom MALDI-TOF mass spectrometry. No significant difference in call rates between cases and controls was seen. Samples successfully genotyped in <95% of markers were excluded from analysis. Markers were excluded if they deviated significantly from Hardy–Weinberg equilibrium (*P* < 0.05, Hardy–Weinberg) or if they had a call rate <95% in the entire cohort.

### Extraction of RNA from a *Mus spretus* × *Mus musculus* F1 backcross

All animal experiments were approved a priori by the UCSF IACUC. Backcross mice were generated by crossing inbred male SPRET/Ei with inbred female FVB/N mice (Jackson Laboratory). Female F1 hybrids were then mated to male FVB/N mice. Lungs from eight-week-old mice were snap-frozen, and RNA was isolated using TRIzol (Invitrogen) according to the manufacturer's instructions. Residual contaminating genomic DNA was removed by DNase treatment (Ambion).

### qRT-PCR TAQMAN analysis

PCR was conducted in triplicate with 20 μL reaction volumes of 1X Taqman buffer (1X Applied Biosystems PCR buffer, 20% glycerol, 2.5% gelatin, 60nM Rox as a passive reference), 5.5 mM MgCl_2_, 0.5 mM each primer, 0.2 μM each deoxynucleotide triphosphate (dNTP), 200 nM probe, and 0.025 unit/μL AmpliTaq Gold (Applied Biosystems) with 5 ng cDNA. A large master mix of the above-mentioned components (minus the primers, probe, and cDNA) was made for each experiment and aliquoted into individual tubes, one for each cDNA sample. cDNA was then added to the aliquoted master mix. The master mix with cDNA was aliquoted into a 384-well plate. The primers and probes were mixed together and added to the master mix and cDNA in the 384-well plate. PCR was conducted on the ABI 7900HT (Applied Biosystems) using the following cycle parameters: 1 cycle of 95° for 10 min and 40 cycles of 95° for 15 s, 60° for 1 min. SDS Analysis (version 2.3) supplied with ABI 7900HT, was used to determine the *Ct* values of each reaction.

*Ct* values were determined for three test and three reference reactions in each sample, averaged, and subtracted to obtain the Δ Ct [Δ Ct = Ct (test locus) − Ct (control locus)]. PCR efficiencies were measured for all custom assays and were =90%. Therefore, relative fold difference was calculated for each primer/probe combination as 2^−ΔCt^ × 100.

Primer probe combinations were either generated in house (mouse *Gus* and rat *Gapdh*):

*Gus*
Forward: CTCATCTGGAATTTCGCCGA
Reverse: GGCGAGTGAAGATCCCCTTC
Probe: CGAACCAGTCACCGCTGAGAGTAATCG
*GapDH*
Forward: TGCACCACCAACTGCTTAG
Reverse: GGATGCAGGGATGATGTTC
Probe: CAGAAGACTGTGGATGGCCCCTC
or purchased as proprietary reagents from ABI-Life Technologies Inc:
*Eng*, mCG18573: **Assay ID:** Mm00468256_m1
*Acvrl1*, mCG16831: **Assay ID:** Mm00437432_m1
*Ptpn14*, mCG19670: **Assay ID:** Mm00501215_m1

## Results

### Genetic variation in the functional *ENG* allele associates with pulmonary AVMs in HHT1

As previously reported, a candidate screen for genetic association to pulmonary AVMs in HHT patients showed that *PTPN14* within the *TGFBM2* locus showed the strongest genetic association to pulmonary AVM incidence in both HHT1 and HHT2 mutation carriers (Benzinou et al., 2012). However, as presented here, additional SNPs within several candidate genes showed suggestive association within the Dutch HHT population (see Supplementary Table S1). Interestingly, in the Dutch HHT cohort, primarily within HHT1, we found evidence of suggestive genetic association between the prevalence of pulmonary AVMs in three out of seven tagSNPs spanning the HHT1 causative gene, *ENG* (*P* = 5.4 × 10^−3^ for the intronic SNP *rs10987746* in HHT1; *GC test*, Supplementary Table S2), and a similar observation was made within the French HHT1 population (*rs*1887266, *P* = 0.005; *rs*10987746; *P* = 0.087; Supplementary Table S3). Our initial analysis did not differentiate between the two alleles of the mutated causative gene. We therefore considered that this observation might be artifactual, driven by linkage disequilibrium between the test SNP and the causative *ENG* mutation. Since most causative HHT mutations are loss of function, we surmised that genetic variants within the mutated gene would be functionally inconsequential whereas genetic variation within the wild type allele, inherited from the unaffected parent, may influence the clinical features of HHT. In order to interrogate this further, we limited our analysis to those Dutch HHT families for whom the genetic phase of the causative mutation could be determined. Pedigree analysis permitted us to elucidate the genetic phase of *ENG rs10987746* with respect to the causative *vs*. wild type *ENG* alleles in 266 HHT1 cases from 80 families. We tested for genetic association between *rs10987746* within the wild type *ENG* allele and the prevalence of pulmonary AVMs in Dutch HHT1, and confirmed the notion that genetic variation within the functional *ENG* allele inherited from the non-affected parent associates with presence of pulmonary AVM in HHT1 (*rs10987746-C* relative risk = 1.3 (1.0018–1.7424) (Table 1).

**Table 1:** **Genetic variation in the wild type ENG allele, inherited from the unaffected parent, associates with presence of pulmonary AVMs in HHT1 patients**.

***rs10987746* genotype of wild type *ENG* allele**	**HHT1 patients with pulmonary AVM**	**HHT1 patients no pulmonary AVM**
T	63 (44.4%)	79 (55.6%)
C	70 (56.5%)	54 (43.5%)

*Relative risk of pulmonary AVMs = 1.3 (95% confidence interval = 1.002–1.742) P = 0.018*.

### Possible genetic association between *ACVRL1* and pulmonary AVMs in HHT2

A similar effect to that seen with *ENG* may occur for the wild type *ACVRL1* allele in HHT2. Indeed, four out of six tagSNPs that span the two adjacent loci *ACVRL1* and *ACVR1B* showed genetic association with pulmonary AVMs in Dutch HHT2 patients (*P* < 0.05; GC test, Supplementary Table S2). However, there were insufficient HHT2 specimens within the Dutch HHT population to undertake genetic phase analysis for *ACVRL1*, and extensive pedigree information was not available for the French cohort, pre-empting an analysis of the effect of variation in the functional *ACVRL1* allele on pulmonary AVM incidence.

### Suggestive genetic association between *EMILIN2* variants and pulmonary AVMs in HHT patients

SNPs within several other candidate genes showed suggestive association (*p < 0.05)* within the Dutch HHT population (see Supplementary Table S1). Of three *EMILIN2* SNPs tested, one showed significant (*P* = 0.023) and one showed a trend toward (*P* = 0.09) association with the presence of pulmonary AVMs in the French HHT validation cohort (Supplementary Table S3). SNPs in other candidate genes with association in the Dutch screen did not validate in the French HHT cohort (not shown). The *EMILIN2* association with pulmonary AVMs was weak, but was found in HHT1 only (not HHT2) in both the French and Dutch cohorts. It could represent a false positive due to issues with multiple testing, thus functional validation remains to be undertaken.

### The pulmonary AVM-protective *ENG* allele associates with higher *ENG* expression in human LB cell lines and trends with higher *PTPN14* expression

**Figure 1:**
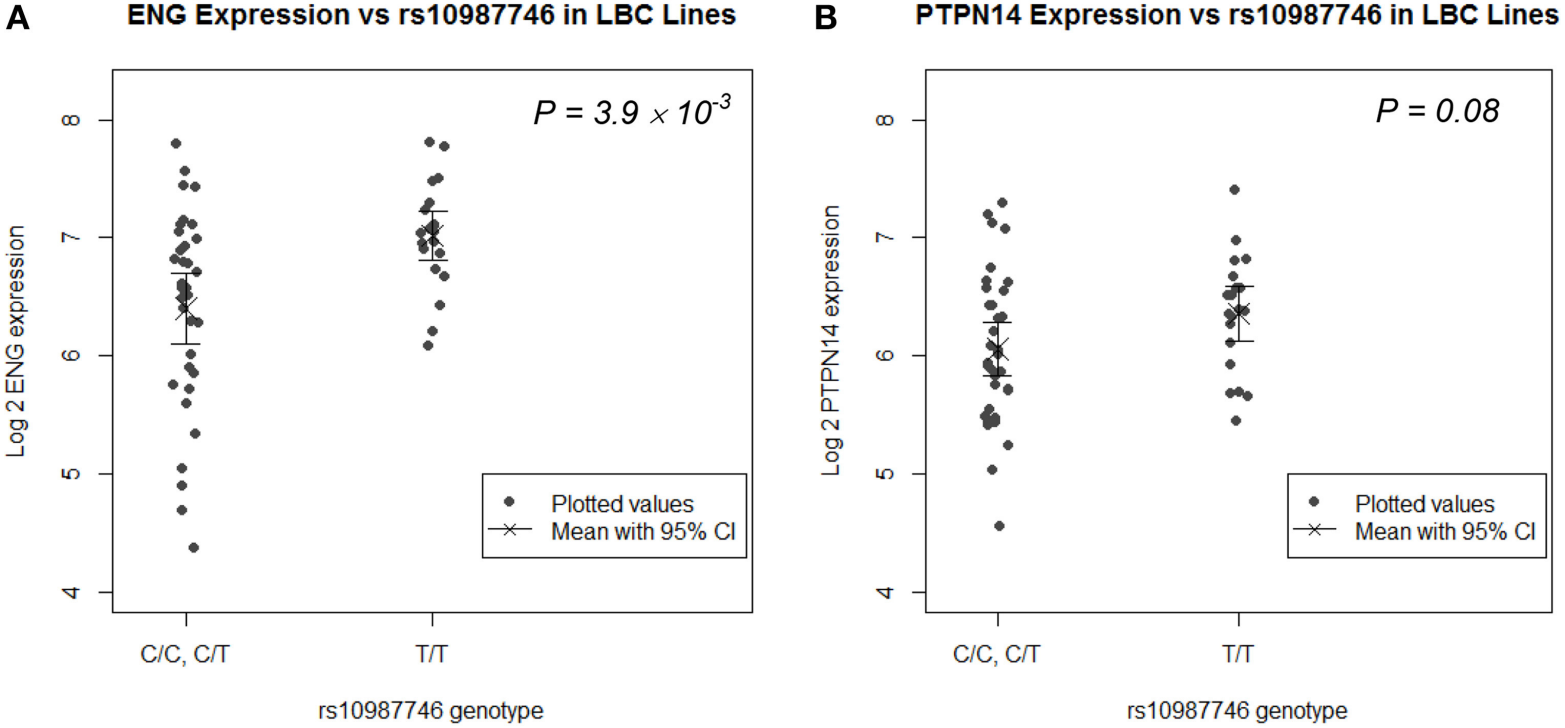
***ENG* SNP *rs10987746* correlates with *ENG* RNA levels in human LB cell lines**. **(A)** *ENG* and **(B)** *PTPN14* expression was assessed according to *rs10987746* genotype in data derived from LB cell lines from Utah residents of Northern and Western European Ancestry from the CEPH collection (CEU) (Cheung et al., 2005). The *rs10987746-C/C* genotype was unusually underrepresented in this panel (*n* = 7), and showed no statistically significant difference in expression from the *rs10987746* -*T/C* genotype, hence these two genotypes were pooled. The mean gene expression and the 95% confidence interval of this mean are plotted for each genotype.

*rs1887266* is a 5′ flanking SNP and *rs10987746* is located within intron 12 near the 3′ end of the gene. These might associate with quantitative differences in *ENG* transcript levels and/or with differential *ENG* splicing resulting in qualitative differences in endoglin protein. We undertook eQTL analysis on publically available genome wide SNP and microarray expression data from 61 CEPH lymphoblastoid (LB) cell lines for which *rs10987746* had been genotyped, to investigate whether this SNP might show correlation to expression of *ENG* message levels. *ENG* mRNA was expressed at low levels in these cell lines (Figure 1A) and the frequency of *rs10987746 –C* homozygosity was exceedingly low (*n* = 7), precluding a statistically robust comparative analysis of *ENG* transcript levels in *rs10987746–CC* genotype *vs*. *rs10987746*-*TC* or *rs10987746*-*TT* genotypes. However, using a dominant model of action for the pulmonary AVM-at-risk allele, *rs10987746-C*, we found significant association with lower *ENG* expression levels (*P* = 4 × 10^−3^, *ANOVA*) (Figure 1A).

Since genetic variation within *PTPN14* is also a genetic risk factor for pulmonary AVMs in HHT1 and HHT2 (Benzinou et al., 2012), we examined association between *ENG-rs10987746*-C and expression of *PTPN14* transcript levels in the human LB dataset. *PTPN14* expression in the CEPH LB cell panel was lower than that of *ENG* and there was a trend toward association between *rs10987746-C* and lower *PTPN14* gene expression, although this did not reach significance (*P* = 0.08, ANOVA; Figure 1B).

### In angiogenically active human lung tumor tissue, but not in normal lung, the pulmonary AVM at-risk *ENG* SNP associates with higher *PTPN14* expression

**Figure 2:**
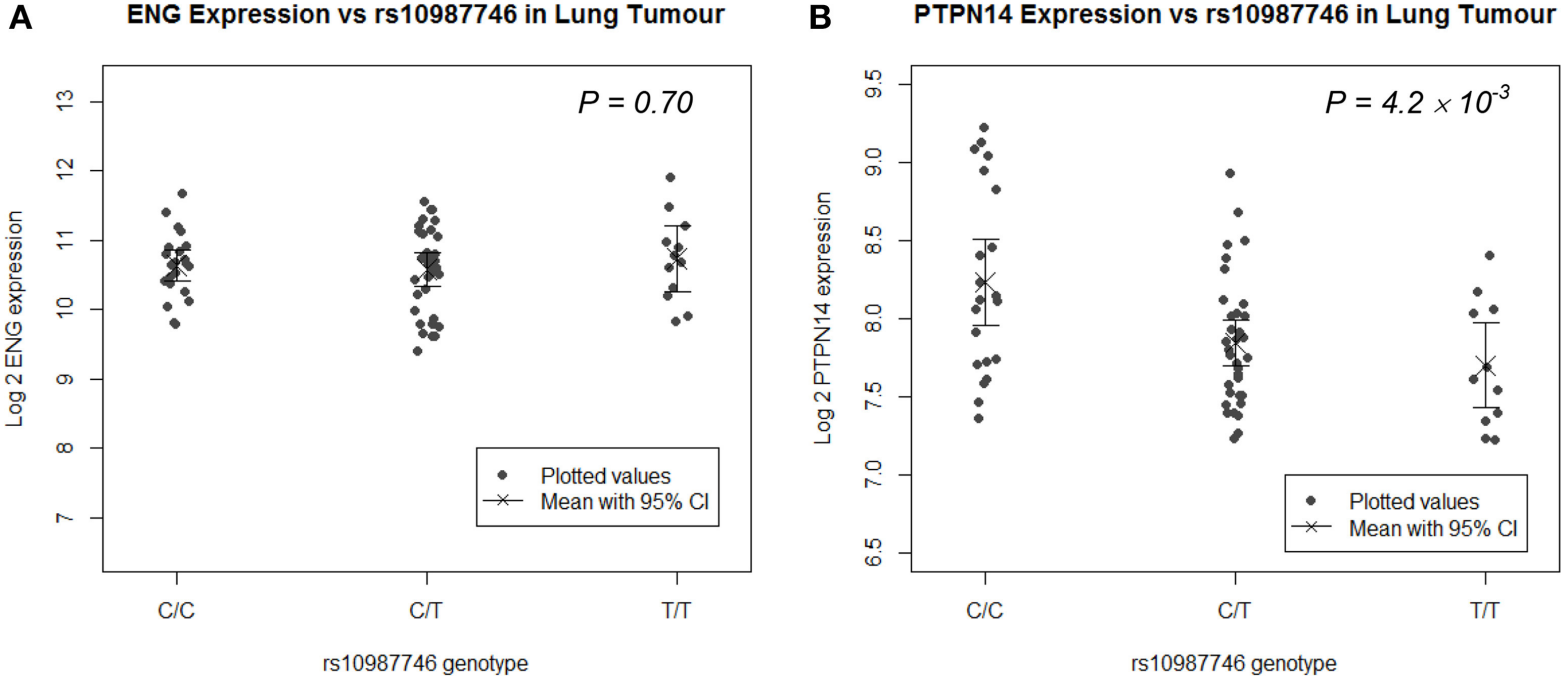
***ENG* SNP *rs10987746* correlates with *PTPN14* but not *ENG* expression levels in human primary pulmonary adenocarcinoma tissue, but not in normal lung**. **(A)** *ENG* and **(B)** *PTPN14* expression was plotted vs. *rs10987746* genotype in RNAs extracted from a panel of 111 primary human lung adenocarcinoma samples **(A,B)** and matched uninvolved lung tissue from the same patients (insignificant see Supplementary Figure S1). The mean gene expression and the 95% confidence interval of this mean are shown for each genotype. Gene expression data was ascertained using Illumina human-6 v2.0 expression beadchip technology (Kim et al., 2013) (GEO GSE32665) and *P-values* (*ANOVA*) were calculated using Tukey's HSD in R.

Since the target organ of interest in this study is lung, we undertook eQTL analysis in a panel of human lung tissues. We genotyped *rs10987746* in genomic DNA and interrogated gene expression levels of *ENG* and *PTPN14* in 111 paired samples of human primary pulmonary adenocarcinomas and matched uninvolved lung tissue from the same individual collected from lung cancer patients who had undergone debulking surgery for their cancer (Kim et al., 2013). We found that, in the uninvolved “normal” human lung tissue, which is angiogenically quiescent, *PTPN14* gene expression levels were high but showed little variation across the panel (≅ 2 fold) precluding robust eQTL analysis (Supplementary Figure S1). On the other hand, in the primary lung adenocarcinoma samples, although *PTPN14* expression levels were lower than in the adjacent unaffected lung, they showed more variation between individuals across the panel (≅ 4 fold). Intriguingly, eQTL analysis showed association between the pulmonary AVM-associated *ENG* SNP, *rs10987746-C*, and higher expression of *PTPN14* in this angiogenically-active adenocarcinoma tissue (*P* = 4 × 10^−3^, Figure 2), whereas *ENG* transcript levels were not correlated with *rs10987746* in either normal or neoplastic lung tissue.

### Expression of *ENG, ACVRL1*, and *PTPN14* are strongly correlated in a genetically diverse panel of mouse lungs

**Figure 3:**
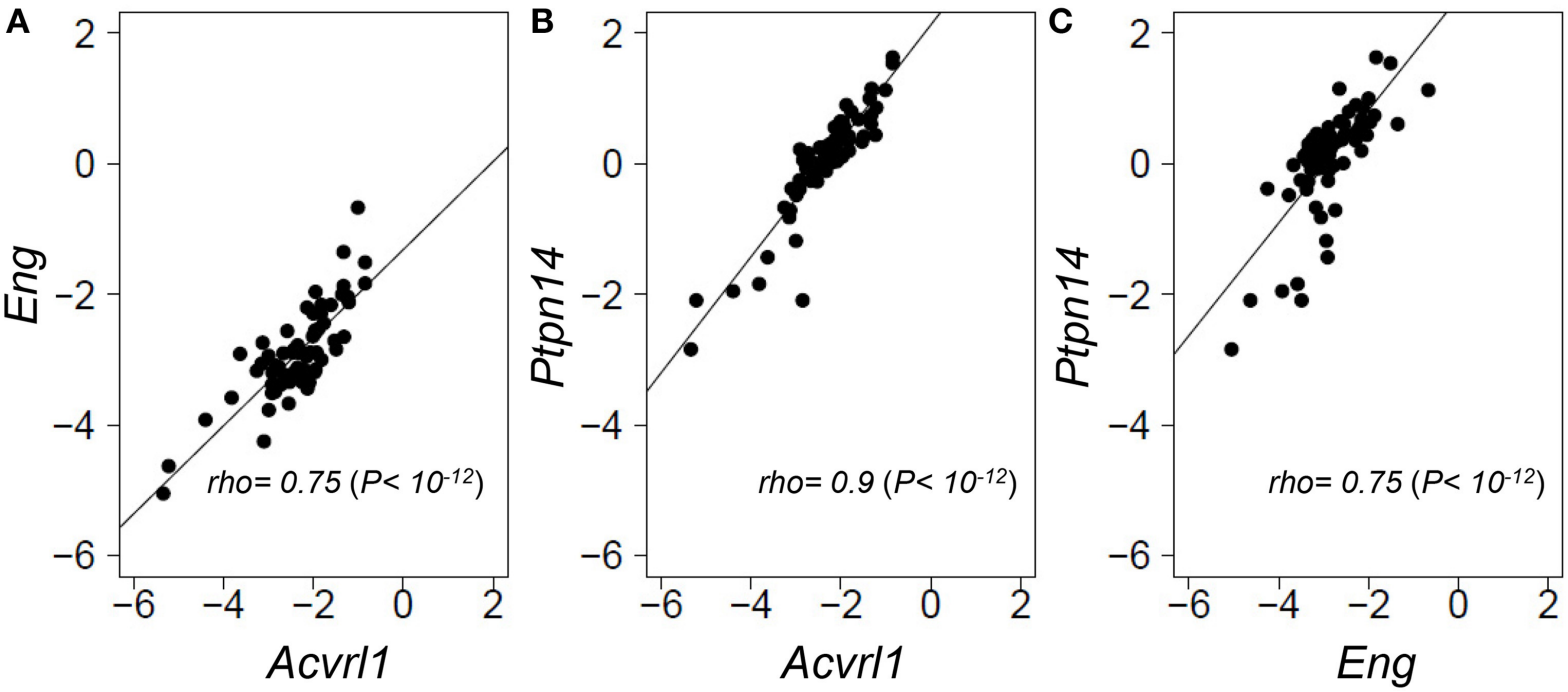
***Eng, Acvrl1*, and *Ptpn14* gene expression are highly correlated in normal mouse lung tissues**. RNA extracted from a panel of 69 wild type healthy mouse lungs from an F1 inter-specific mouse backcross between *Mus spretus* and *Mus musculus* FVB/Ola, was subjected to TAQMAN qRT-PCR analysis with five probes, *Eng, Acvrl1, Ptpn14, Gus*, and *Gapdh*. **(A–C)** show pair-wise correlations for expression of the two probes indicated in each of the 69 samples. *Eng, Acvrl1*, and *Ptpn14*, expression are normalized against *Gapdh*. The data showed a similar strong correlation when normalized against *Gus* (not shown). Non-parametric Spearman Rank correlations shown were calculated in R.

As *PTPN14* gene expression levels were relatively homogeneous across the panel of normal angiogenically quiescent human lung tissues, we turned to a genetically heterogeneous mouse model to interrogate possible correlations between gene expression of the two HHT causative genes, *Eng* and *Acvrl1*, with the pulmonary AVM modifier, *Ptpn14*. An F1 inter-specific mouse backcross between the two inbred mouse species, *Mus spretus* and *Mus musculus* FVB/Ola provides even greater genetic heterogeneity than seen within the human population, such that individual genes show a wider dynamic range of expression levels between individual mice, thus permitting more powerful expression correlation analysis (Quigley et al., 2009; Kim et al., 2013; Sjolund et al., 2014). We quantified relative expression levels of *Eng, Acvrl1*, and *Ptpn14* transcripts across this inter-specific normal mouse lung panel and found that the three test genes showed highly correlated expression levels with each other (Spearman's *rho* values from 0.75 to 0.9, *P* = 1 × 10^−12^; Figure 3). This suggests that *Eng, Acvrl1*, and *Ptpn14* may all be expressed in the same lung cell type (Quigley et al., 2009; Kim et al., 2013), presumably endothelial, and that they are possibly co-regulated within that cell type (Sjolund et al., 2014). However, the data cannot exclude the possibility that tight expression correlation is due to crosstalk between two intimately interacting cell types.

## Discussion

The identification of modifier genes can be informative for elucidating possible molecular mechanisms regulating variable penetrance and expressivity of clinical symptoms of disease. In the current study we show that genetic variation in the wild type *ENG* allele, inherited from the unaffected parent, modifies HHT clinical outcome in terms of presence of pulmonary AVMs in HHT1. We found that the pulmonary AVM-at-risk allele, *ENG-rs10987746-C*, associates with a small but significant reduction in *ENG* transcript levels in a panel of human LB cell line data, and we demonstrate significant expression interaction between *ENG, ACVRL1*, and the pulmonary AVM modifier, *PTPN14*, by eQTL analysis and by expression correlation analysis of mouse and human lung tissue *in vivo*. We also report suggestive association between SNPs in *EMILIN2*, which encodes the extracellular matrix protein, elastin microfibril interfacer 2, in two independent cohorts of HHT1 patients from Netherlands and France.

There have been previous reports of genetic association between SNPs in *ACVRL1* and *ENG* with sporadic brain AVMs in North American and Northern European Caucasians. Pawlikowska et al. (2005) demonstrated association of sporadic brain AVMs with *ACVRL1-rs2071219*, and marginal association with *ENG-rs16930129* (*P* = 0.002) and *ENG-rs10987759* (*P* = 0.09) in 177 sporadic brain AVM patients vs. 129 healthy controls. Simon et al. (2006) replicated the association between *ACVRL1-rs2071219* with brain AVMs or dural arteriovenous fistulas in a Northern European cohort, but were unable to show such association with *ENG* SNPs. Conversely, *ACVRL1-rs2071219* association with sporadic brain AVMs was not reproduced in a more recent study of 143 Dutch Caucasian patients vs. 360 ethnically matched healthy volunteers (Boshuisen et al., 2013). Within HHT, Boshuisen et al. (2013) screened for genetic association between *ENG* SNPs and presence of brain AVMs within the Dutch HHT1 population by matched pair analysis of 24 HHT1 patients with brain AVMs vs. 24 HHT1 relatives with no brain AVM, but they were unable to show genetic association, possibly due to small sample sizes of the brain AVM-positive HHT1 population. In the current study, we genotyped larger numbers of HHT1 patients and focused on pulmonary AVMs which is more common than brain AVM. We were able to screen for genetic association to the wild type *ENG* allele in 266 affected HHT1 individuals from 35 families, for whom we could deduce the phase of the wild type *ENG* SNP. We found weak but significant genetic association between the inheritance of a wild type *ENG rs10987746-C* allele and increased risk of pulmonary AVMs in HHT1.

Here we complemented our previous HHT studies implicating *PTPN14* as a genetic modifier of pulmonary AVMs by demonstrating strong expression correlation between *Eng, Acvrl1*, and *Ptpn14* transcript levels in normal wild type mouse lung tissues. Our previous studies utilizing correlation gene expression network analysis of interspecific mouse backcrosses have demonstrated the power of this technique to define not only cell-type-specific co-expression, but to identify genes whose protein products show direct physical or functional interactions within tissues (Quigley et al., 2009; Sjolund et al., 2014). The strong correlation between expression of these three genes, together with expression of other endothelial-specific genes (data not shown), suggests that in normal mouse lung *Ptpn14, Eng, and Acvrl1* are predominantly co-expressed within the same cell type, namely endothelial. However, there are currently no PTPN14-specific antibodies that perform well for immunohistochemical analysis to test this possibility.

We found that the pulmonary AVM-at-risk allele *rs10987746-C* associated with a slight but significantly lower level of *ENG* RNA in a human LB cell line panel, providing supporting evidence that genetic variation within this gene may influence its own expression. Conversely, in a panel of RNAs extracted from human primary lung adenocarcinomas and from adjacent normal lung tissue, there was no correlation between *ENG rs10987746* and *ENG* expression level. This may, in part, be due to the technical limitations of working with primary human tissues compared to cell lines, and due to differences in the technological platforms used for gene expression analysis in the two experiments. However, in the human lung tumor tissues *rs10987746* did show correlation with expression levels of *PTPN14*, despite lack of association with *ENG* transcript levels. A possible explanation is that this SNP is associated with differential post-transcriptional or translational control of endoglin levels that subsequently affect *PTPN14* expression. *rs10987746* lies within an antisense transcript showing 300 nt complementarity to the *ENG* mRNA that might contribute to regulation of *ENG* in either *cis* or *trans*. Additionally, *rs10987746* is proximal to the penultimate *ENG* exon which is subject to differential splicing to generate two structurally distinct and functionally antagonistic endoglin protein isoforms differing within their intracellular domains (Bellon et al., 1993; Lopez-Novoa and Bernabeu, 2010). Whether or not *rs10987746* associates with differential *ENG* splicing, remains to be tested. Overall, however, we conclude that correlations between *ENG, ALK1*, and *PTPN14* expression in both mouse and human lung tissues, support our previous *in vitro* studies of reciprocal regulation between *ACVRL1* and *PTPN14* in primary human endothelial cells, and genetic association between *PTPN14* and risk of pulmonary AVMs in HHT (Benzinou et al., 2012).

Since publishing our findings on *PTPN14* involvement in HHT (Benzinou et al., 2012), several papers have demonstrated that, in epithelial cells, PTPN14 is a negative regulator of the transcriptional cofactors, Yap, and Taz (Huang et al., 2012; Liu et al., 2012; Wang et al., 2012; Wilson et al., 2014). These proteins are targets of the Hippo kinase pathway, originally identified as a master regulator of cell and organ size, mediator of contact inhibition and regulator of stem cell maintenance (Barry and Camargo, 2013; Hiemer and Varelas, 2013). More recently, Hippo signaling has been found to be a major regulator of mechanotransduction in both epithelial and endothelial cells (Dupont et al., 2011; Sansores-Garcia et al., 2011; Wada et al., 2011; Zhao et al., 2012; Aragona et al., 2013). Intriguingly, *EMILIN2*, which showed suggestive association with pulmonary AVMs and which is known to regulate angiogenesis, has also been reported to regulate Taz activity (Marastoni et al., 2014), suggesting some commonality in the downstream pathways of two pulmonary AVM modifier genes.

Blood vessels, in particular, are subject to constant shear stress and remodeling, suggesting that disruption of endothelial cell signaling pathways that regulate mechanotransduction within the vascular bed may contribute to defects in vascular remodeling and AVM susceptibility (Roman and Pekkan, 2012). The Roman group previously demonstrated an important link between the fish orthologue of *ACVRL1, alk1*, and regulation of mechanotransduction when using the zebrafish as a model system. They demonstrated not only that *alk1* expression in arterial endothelial cells requires blood flow, but that under normal shear stress conditions a subset of flow-responsive genes are dysregulated in *alk1* mutant arterial endothelial cells (Corti et al., 2011). They proposed a model whereby blood flow, under normal conditions, not only activates *alk1* expression, but delivers cardiac-derived Bmp10 to activate an Alk1-responsive (flow-responsive) gene expression program that limits nascent arterial caliber (Laux et al., 2013). The interaction between PTPN14, endoglin, and ALK1 in mouse and human tissues provides another molecular link between the HHT causative genes and the mechanotransduction pathway. It will be important to elucidate the detailed molecular mechanisms regulating these interactions in endothelial cells, particularly within the lung, since this might lead to more rational choices for HHT therapy.

## Author contributions

All authors made substantial contributions to the conception or design of the work or contributed to acquisition, analysis, or interpretation of data and assisted in drafting or revising the manuscript, and approved the final version and agree to be accountable for all aspects of the work, ensuring that questions related to the accuracy or integrity of any part of the work are appropriately investigated and resolved.

### Conflict of interest statement

The authors declare that the research was conducted in the absence of any commercial or financial relationships that could be construed as a potential conflict of interest.
