# Supplementary material for: Genetic variation in the functional ENG allele inherited from the non-affected parent associates with presence of pulmonary arteriovenous malformation in hereditary hemorrhagic telangiectasia 1 (HHT1) and may influence expression of PTPN14
Source: Front Genet. 2015 Mar 12;6:67. doi: 10.3389/fgene.2015.00067 (PMC4357294; doi:10.3389/fgene.2015.00067)
Supplement: Supplementary file 1 [file Table1.PDF]

Table S1: SNPs (genes) with suggestive genetic association with the presence of pulmonary AVMs in HHT mutations carriers.

SNP	Gene	Chr	Chr position	Gene Selection	Cohort	HHT1 and HHT2 families					HHT1 families					HHT2 families				
						P-value	FREQ	N	FREQ	N	P-value	FREQ	N	FREQ	N	P-value	FREQ	N	FREQ	N
rs5019497	***TGFBR3***	1	92038221	Candidate Gene	Original Scan (OS)	**0.0085**	0.52	A	0.48	C	0.11	0.53	A	0.47	C	**0.0096**	0.54	A	0.46	C
					OS + Extension	**0.0063**	0.52	A	0.48	C	0.1	0.53	A	0.47	C	**0.0048**	0.54	A	0.46	C
rs10493859	***TGFBR3***	1	92046232	Candidate Gene	Original Scan (OS)	**0.0074**	0.3	A	0.7	C	0.18	0.28	A	0.72	C	**0.0086**	0.3	A	0.7	C
					OS + Extension	0.012	0.3	A	0.7	C	0.23	0.28	A	0.72	C	0.014	0.3	A	0.7	C
rs3002297	***PTPN14****	1	212602684	*TGFBM2*	Original Scan (OS)	**0.0097**	0.75	G	0.25	A	0.012	0.77	G	0.23	A	0.15	0.68	G	0.32	A
					OS + Extension	0.029	0.75	G	0.25	A	0.03	0.77	G	0.23	A	0.23	0.7	G	0.3	A
rs3002300	***PTPN14****	1	212605233	*TGFBM2*	Original Scan (OS)	**0.008**	0.77	T	0.23	A	0.016	0.79	T	0.21	A	0.1	0.72	T	0.28	A
					OS + Extension	**0.00018**	0.77	T	0.23	A	**0.0017**	0.79	T	0.21	A	0.1	0.72	T	0.28	A
rs2936017	***PTPN14****	1	212615305	*TGFBM2*	Original Scan (OS)	**0.0089**	0.77	A	0.23	G	0.017	0.79	A	0.21	G	0.11	0.72	A	0.28	G
					OS + Extension	**0.0002**	0.77	A	0.23	G	**0.0094**	0.79	A	0.21	G	0.013	0.72	A	0.28	G
rs2936018	***PTPN14****	1	212627975	*TGFBM2*	Original Scan (OS)	**0.00095**	0.79	C	0.21	T	**0.0097**	0.8	C	0.2	T	0.051	0.75	C	0.25	T
					OS + Extension	**0.000028**	0.79	C	0.21	T	**0.0012**	0.8	C	0.2	T	**0.0091**	0.75	C	0.25	T
rs700024	***USH2A****	1	214509837	*TGFBM2*	Original Scan (OS)	0.014	0.88	C	0.12	G	**0.0072**	0.88	C	0.12	G	0.72	0.9	C	0.1	G
					OS + Extension	**0.0016**	0.88	C	0.12	G	**0.0048**	0.88	C	0.12	G	0.46	0.9	C	0.1	G
rs1891467	***TGFB2****	1	216646608	Candidate Gene	Original Scan (OS)	**0.0084**	0.77	A	0.23	G	0.013	0.76	A	0.24	G	0.67	0.8	A	0.2	G
					OS + Extension	**0.0055**	0.77	A	0.23	G	0.011	0.76	A	0.24	G	0.37	0.79	A	0.21	G
rs10495565	***ADAM17****	2	9603891	*TGFBM3*	Original Scan (OS)	0.029	0.28	G	0.72	A	0.011	0.28	G	0.72	A	0.87	0.30	G	0.70	A
					OS + Extension	**0.007**	0.29	G	0.71	A	0.017	0.29	G	0.71	A	0.28	0.62	G	0.38	A
rs12474540	***ADAM17****	2	9616764	*TGFBM3*	Original Scan (OS)	0.026	0.37	C	0.63	T	0.018	0.36	C	0.64	T	0.95	0.20	C	0.80	T
					OS + Extension	**0.01**	0.37	C	0.63	T	0.03	0.38	C	0.62	T	0.22	0.20	C	0.80	T
rs3769550	***LTBP1***	2	33436550	Candidate Gene	Original Scan (OS)	**0.00097**	0.21	G	0.79	C	**0.0024**	0.2	G	0.8	C	0.2	0.24	G	0.76	C
					OS + Extension	**0.00035**	0.21	G	0.79	C	**0.0018**	0.2	G	0.8	C	0.14	0.24	G	0.76	C
rs2237436	***INHBA***	7	41697456	Candidate Gene	Original Scan (OS)	0.028	0.37	C	0.63	G	0.11	0.38	C	0.62	G	0.13	0.36	C	0.64	G
					OS + Extension	0.035	0.37	C	0.63	G	0.12	0.38	C	0.62	G	0.16	0.35	C	0.65	G
rs2237432	***INHBA***	7	41701559	Candidate Gene	Original Scan (OS)	0.027	0.25	C	0.75	T	0.07	0.24	C	0.76	T	0.13	0.24	C	0.76	T
					OS + Extension	**0.0094**	0.25	C	0.75	T	0.056	0.24	C	0.76	T	0.11	0.24	C	0.76	T
rs2877098	***INHBA***	7	41709819	Candidate Gene	Original Scan (OS)	**0.0011**	0.32	T	0.68	C	0.021	0.32	T	0.68	C	0.021	0.29	T	0.71	C
					OS + Extension	**0.0013**	0.32	T	0.68	C	0.024	0.32	T	0.68	C	0.026	0.29	T	0.71	C
rs10987746	***ENG***	9	129619914	Causal gene	Original Scan (OS)	**0.0054**	0.46	C	0.54	T	0.023	0.48	C	0.52	T	0.27	0.38	C	0.62	T
					OS + Extension	0.012	0.46	C	0.54	T	0.011	0.48	C	0.52	T	0.24	0.38	C	0.62	T
rs7865146	***5' ENG***	9	129659458	Causal Gene	Original Scan (OS)	0.02	0.62	T	0.38	C	0.12	0.52	T	0.48	C	0.43	0.72	T	0.27	C
					OS + Extension	0.012	0.62	T	0.38	C	0.08	0.52	T	0.48	C	0.31	0.72	T	0.27	C
rs1887266	***5' ENG, 3' AK1***	9	129664943	Causal Gene	Original Scan (OS)	0.156	0.07	A	0.93	G	0.06	0.07	A	0.93	G	0.75	0.22	A	0.78	G
rs11169953	***ACVRL1***	12	50590666	Causal Gene	Original Scan (OS)	0.035	0.69	C	0.31	T	0.12	0.68	C	0.32	T	0.25	0.7	C	0.3	T
					OS + Extension	0.028	0.69	C	0.31	T	0.09	0.68	C	0.32	T	0.21	0.7	C	0.3	T
rs10783485	***ACVRL1/ACVR1B***	12	50622274	Causal Gene	Original Scan (OS)	**0.0046**	0.69	G	0.65	T	0.25	0.34	G	0.66	T	**0.0013**	0.5	G	0.5	T
					OS + Extension	**0.0022**	0.69	G	0.65	T	0.25	0.34	G	0.66	T	**0.0008**	0.5	G	0.5	T
rs11610143	***ACVR1B***	12	50635338	Candidate Gene	Original Scan (OS)	**0.0048**	0.2	G	0.8	C	0.49	0.2	G	0.8	C	**0.0001**	0.38	G	0.62	C
					OS + Extension	**0.005**	0.2	G	0.8	C	0.031	0.2	G	0.8	C	**0.00004**	0.38	G	0.62	C
rs12809597	***ACVR1B***	12	50642590	Candidate Gene	Original Scan (OS)	0.016	0.73	T	0.23	G	0.05	0.736	T	0.26	G	0.31	0.69	T	0.31	G
rs623561	***EMILIN2***	18	2862586	Candidate Gene	Original Scan (OS)	**0.0043**	0.57	A	0.43	G	**0.0039**	0.58	A	0.42	G	0.36	0.56	A	0.44	G
					OS + Extension	**0.0091**	0.57	A	0.43	G	**0.009**	0.58	A	0.42	G	0.37	0.56	A	0.44	G
rs583523	***EMILIN2***	18	2864156	Candidate Gene	Original Scan (OS)	0.034	0.33	G	0.67	A	0.052	0.33	G	0.67	A	0.42	0.38	G	0.62	A
					OS + Extension	0.036	0.33	G	0.67	A	0.057	0.33	G	0.67	A	0.45	0.37	G	0.63	A
rs642887	***EMILIN2***	18	2864408	Candidate Gene	Original Scan (OS)	0.029	0.86	G	0.14	A	**0.0074**	0.86	G	0.14	A	0.47	0.92	G	0.08	A
					OS + Extension	**0.0089**	0.86	G	0.14	A	**0.0057**	0.86	G	0.14	A	0.71	0.91	G	0.09	A
rs12454179	***EMILIN2***	18	2865255	Candidate Gene	Original Scan (OS)	**0.0093**	0.74	G	0.26	A	**0.0081**	0.75	G	0.25	A	0.32	0.76	G	0.24	A
					OS + Extension	**0.013**	0.74	G	0.26	A	0.017	0.75	G	0.25	A	0.45	0.76	G	0.24	A

* Previously published in Benzinou 2012 and Kawasaki 2014. P values < 0.01 are shown in bold.
