# Supplementary material for: Genetic variation in the functional ENG allele inherited from the non-affected parent associates with presence of pulmonary arteriovenous malformation in hereditary hemorrhagic telangiectasia 1 (HHT1) and may influence expression of PTPN14
Source: Front Genet. 2015 Mar 12;6:67. doi: 10.3389/fgene.2015.00067 (PMC4357294; doi:10.3389/fgene.2015.00067)
Supplement: Supplementary file 2 [file Table2.PDF]

Supplementary Table S2. Genetic association to *ENG* and *ACVRL1* SNPs in Dutch HHT by Gamete Competition analysis

		Chr position		All HHT							HHT1							HHT2						
SNP	*GENE*	(GRCh38)	MAF	P Value	Tau	allele freq (a)	allele a	Tau	allele freq (b)	allele b	HHT1	Tau	allele freq (a)	allele a	Tau	allele freq (b)	allele b	HHT2	Tau	allele freq (a)	allele a	Tau	allele freq (b)	allele b
rs10987746	***ENG***	**9:127817814**	**0.48**	**0.005**	**0.71**	**0.46**	**C**	**1**	**0.54**	**T**	**0.02**	**0.70**	**0.48**	**C**	**1**	**0.52**	**T**	**0.27**	**1.04**	**0.38**	**C**	**1**	**0.62**	**T**
rs3739817	*ENG*	9:127824409	0.05	1.00	1.00	0.08	T	1	0.92	C	0.62	1.13	0.07	T	1	0.93	C	0.54	1.29	0.16	T	1	0.84	C
rs11792480	*ENG*	9:127835846	0.17	0.44	0.91	0.35	T	1	0.65	C	0.91	0.98	0.38	T	1	0.62	C	0.05	1.04	0.44	T	1	0.56	C
rs4836585	*ENG*	9:127850104	0.18	0.73	0.94	0.12	G	1	0.89	T	0.48	0.87	0.13	G	1	0.87	T	0.80	1.02	0.39	G	1	0.61	T
rs10987759	*ENG*	9:127856098	0.1	0.26	0.78	0.08	C	1	0.92	G	0.16	0.69	0.08	C	1	0.92	G	1.00	1.10	0.12	C	1	0.88	G
rs7865146	*5'ENG, 3'AK1*	9:127857358	0.42	**0.012**	1.35	0.38	C	1	0.62	T	0.12	0.80	0.48	C	1	0.52	T	0.44	1.06	0.28	C	1	0.72	T
rs1887266	*5'ENG, 3'AK1*	9:127862843	0.09	0.16	0.73	0.07	A	1	0.93	G	0.06	0.61	0.08	A	1	0.92	G	0.75	0.90	0.22	A	1	0.78	G
rs3759178	*5'ACVRL1*	12:51905475	0.38	0.21	0.86	0.43	G	1	0.57	T	0.54	0.92	0.43	G	1	0.57	T	0.20	1.13	0.12	G	1	0.88	T
rs3782479	*5'ACVRL1*	12:51908034	0.08	0.12	1.45	0.07	A	1	0.93	C	0.43	1.26	0.07	A	1	0.93	C	0.14	0.82	0.44	A	1	0.56	G
rs11169953	*5'ACVRL1*	12:51910615	0.4	**0.035**	1.33	0.31	T	1	0.69	C	0.12	1.27	0.32	T	1	0.68	C	0.25	1.35	0.30	T	1	0.70	C
rs10783485	*3'ACVRL1/5'ACVR1B*	12:51942223	0.43	**0.005**	0.69	0.35	T	1	0.65	G	0.25	0.83	0.34	T	1	0.66	G	**0.0013**	1.43	0.50	T	1	0.50	G
rs11610143	*3'ACVRL1/5'ACVR1B*	12:51955287	0.24	**0.005**	0.65	0.20	G	1	0.80	C	0.49	0.88	0.20	G	1	0.80	C	**0.0001**	1.05	0.38	G	1	0.62	C
rs12809597	*ACVR1B*	12:51962539	0.18	**0.016**	0.73	0.34	G	1	0.66	T	**0.05**	0.74	0.32	G	1	0.68	T	0.31	1.36	0.29	G	1	0.71	T
