# Supplementary material for: Genetic variation in the functional ENG allele inherited from the non-affected parent associates with presence of pulmonary arteriovenous malformation in hereditary hemorrhagic telangiectasia 1 (HHT1) and may influence expression of PTPN14
Source: Front Genet. 2015 Mar 12;6:67. doi: 10.3389/fgene.2015.00067 (PMC4357294; doi:10.3389/fgene.2015.00067)
Supplement: Supplementary file 3 [file Table3.PDF]

Supplementary Table S3. SNPs validated in French HHT population

					HHT all	Minor		Major		HHT1	Minor		Major		HHT2	Minor		Major	
					P Value	***Allele Freq***	N	***Allele Freq***	N	***P Value***	***Allele Freq***	N	***Allele Freq***	N	***P Value***	***Allele Freq***	N	***Allele Freq***	N
1	FRENCH	*rs623561*	chr18:2862586	*EMILIN2*	0.10	0.35	G	0.65	A	**0.023**	0.34	G	0.66	A	0.38	0.38	G	0.62	A
	DUTCH				**0.009**	0.43	G	0.57	A	**0.009**	0.42	G	0.58	A	0.38	0.20	G	0.80	A
2	FRENCH	*rs642887*	chr18:2864408	*EMILIN2*	0.81	0.12	A	0.88	G	0.99	0.12	A	0.88	G	0.07	0.16	A	0.84	G
	DUTCH				**0.006**	0.13	A	0.87	G	**0.014**	0.15	A	0.85	G	0.54	0.74	A	0.26	G
3	FRENCH	*rs12454179*	chr18:2865255	*EMILIN2*	0.09	0.26	A	0.74	G	0.09	0.25	A	0.75	G	0.59	0.25	A	0.75	G
	DUTCH				**0.013**	0.26	A	0.74	G	**0.017**	0.25	A	0.75	G	0.45	0.19	A	0.81	G
4	FRENCH	*rs10987746*	chr9:129619914	*FPGS, ENG*	0.09	0.44	C	0,56	T	0.27	0,45	C	0.55	T	0.60	0.49	C	0.51	T
	DUTCH				**0.005**	0.46	C	0.54	T	**0.023**	0.48	C	0.52	T	0.27	0.38	C	0.62	T
5	FRENCH	*rs1887266*	chr9:129664943	*ENG, AK1*	**0.005**	0.07	A	0.93	G	**0.035**	0.07	A	0.93	G	0.55	0.06	A	0.94	G
	DUTCH				0.16	0.07	A	0.93	G	0.06	0.08	A	0.92	G	0.75	0.22	A	0.78	G

$p < 0.1$

$p < 0.05$
