# Supplementary figures and images for: Genetic variation in the functional ENG allele inherited from the non-affected parent associates with presence of pulmonary arteriovenous malformation in hereditary hemorrhagic telangiectasia 1 (HHT1) and may influence expression of PTPN14
Source: Front Genet. 2015 Mar 12;6:67. doi: 10.3389/fgene.2015.00067 (PMC4357294; doi:10.3389/fgene.2015.00067)

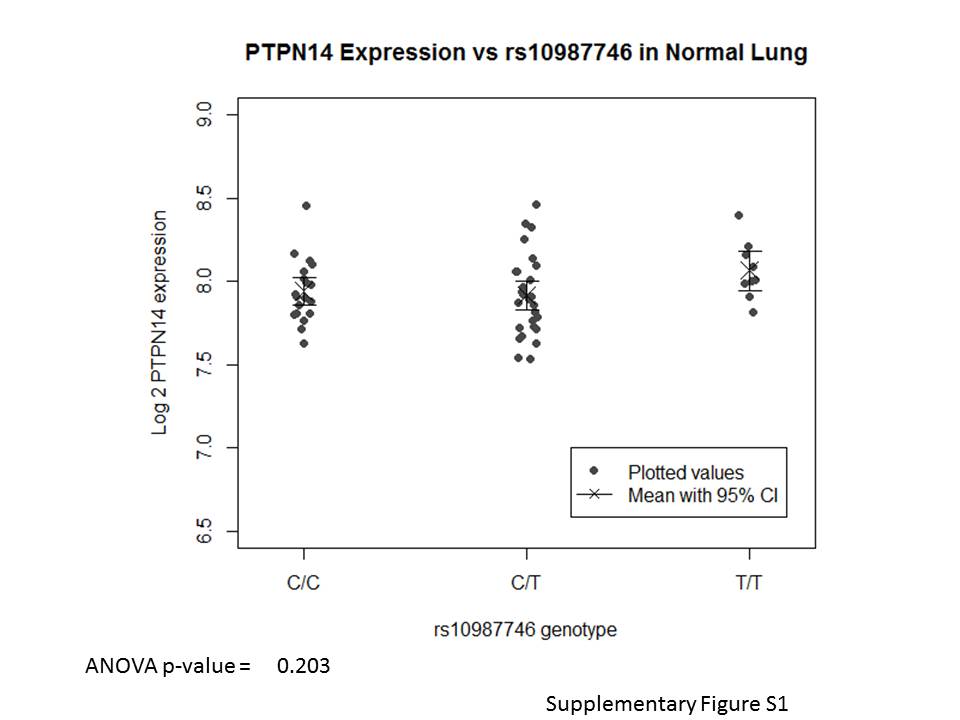

Supplement: Supplementary file 4 [file Image1.JPEG]
